# Comparison of the Short-Term Effect between Iontophoresis and Radial Extracorporeal Shockwave Therapy in the Treatment of Plantar Fasciitis: A Randomized Controlled Trial

**DOI:** 10.3390/healthcare12121223

**Published:** 2024-06-19

**Authors:** Manuel Pabón-Carrasco, Manuel Coheña-Jiménez, Ana Juana Pérez-Belloso, José Algaba-del-Castillo, Rocío Cáceres-Matos, Aurora Castro-Méndez

**Affiliations:** Faculty of Nursing, Physiotherapy and Podiatry, University of Sevilla, 41009 Sevilla, Spain; mpabon2@us.es (M.P.-C.); aperez30@us.es (A.J.P.-B.); algaba@us.es (J.A.-d.-C.); rcaceres3@us.es (R.C.-M.)

**Keywords:** fasciitis, plantar, iontophoresis, extracorporeal shockwave therapy, clinical trial

## Abstract

Conservative treatments for plantar fasciitis have different levels of effectiveness, so it is necessary to personalize the therapeutic modality that improves the patients’ symptoms. Methods: A double-blinded randomized clinical trial was designed to evaluate the short-term efficacy of a physical treatment in chronic plantar fasciitis, namely iontophoresis, compared with radial shockwave therapy. Heel pain, health status using the EuroQol-5D questionnaire, and fascia thickness measured with ultrasound were evaluated. In total, 127 patients were randomly selected for group A and treated with iontophoresis therapy (lidocaine 0.4% and dexamethasone 0.5%), or for group B, in which they were treated with radial shockwave therapy (EWST). Measurements were taken at baseline and at follow-up during the 5 weeks of the study. Results: Statistically significant differences were observed to the shockwave therapy group in respect to the final fascia thickness, and the VAS scale (*p* = 0.001). The differences between groups A and B showed that the shockwave group follow-up after 3 weeks experienced complete pain remission (1.0 ± 0.9; 95%CI 0.8–1.2) and after the 6-week follow-up, complete pain remission of plantar fasciitis was observed for both therapies. Patients had a better perception of the use of EWST at the end of the treatment, although in both groups it was satisfactory (*p* = 0.001). Conclusions: The results of this study showed a shorter-term effectiveness of shockwave treatment compared with the use of iontophoresis. However, both techniques were effective in satisfactorily reducing pain in this short period.

## 1. Introduction

Plantar fasciitis (PF) is the most common cause of heel pain in adults, affecting 10% of the general population [1]. PF or plantar fasciosis is a degeneration of the plantar fascia that generates an inflammatory reaction, secondary to the biomechanical stress to which the plantar fascia is subjected, and it is a degenerative irritation of the plantar fascia origin at the medial calcaneal tuberosity of the heel and its surrounding perifascial structures. It is a degenerative process of the plantar fascia secondary to repetitive microtrauma; several etiological factors, such as obesity, sex, physical exercise, or biomechanical disorders, can be related to it [2]. The symptoms that patients report are medial plantar pain in weight, which increases during the first steps they take in the morning and improves at rest. This heel pain worsens the quality of life of patients. For this reason, a detailed musculoskeletal examination must be performed on the lower extremities to identify the clinical symptoms of the pain that the patient reports and that is reproducible [1,2].

Different diagnostic evaluations of plantar fasciitis have been described that include X-rays, ultrasounds, resonance, and scintigraphy, among others. Although the diagnosis of this chronic degenerative process is simple and clinical, a differential diagnosis with other pathologies (skeletal origin, soft tissue origin, or neurogenic origin) needs to be carried out in order to provide adequate treatment. The diagnosis is based on the patient’s symptoms, and after 8 weeks of pain it is to be considered chronic PF; this is also the case if the ultrasound image shows a degenerate, hypoechogenic (low brightness intensity of the image of the plantar fascia related to ultrasound waves and associated with pain), and thick fascia [3].

There are different treatment modalities for plantar fasciitis that must be taken into account in the therapeutic treatment plan, starting with conservative measures and ending with long-term surgical management, such as Achilles’ lengthening [3]. The conservative treatments are the first option, and these include plantar orthoses [4,5], muscle stretching [6,7], functional taping [8], radial extracorporeal shockwave therapy (rESWT) [9,10,11], laser therapy [12], systemic pharmacological therapy [13], and percutaneous injection [14,15]. Some of them are an important aid when the patient needs another definitive treatment (plantar orthoses or stretching), so sometimes they are relevant as provisional treatment to improve pain quickly, although surgical treatment is considered when conservative treatments have failed [16,17,18]. Therefore, conservative treatments for PF are sometimes not effective, so it is important to research new treatments that can help, and corticosteroids are sometimes administered response it quickly effects on the pain. Usually, this drug is administered by infiltration.

Really, the efficacy of the application of iontophoresis at the foot level has not been sufficiently explored, and there is a lack of scientific evidence, to the authors’ knowledge. This treatment is based on the use of low-voltage galvanic stimulation to conduct a drug into tissues, and it is an alternative to replace the local infiltration. However, the use of iontophoresis has promising results when pain is sometimes an important symptom that can affect the daily activities of the patient. Iontophoresis is used in various lower extremity related pathologies, such as hyperhidrosis, tendinopathies, and musculoskeletal pathologies involving certain social restrictions, mobility difficulties, and pain [19].

A wide variety of conservative management techniques are available for the management of plantar fasciitis. The administration of drugs by iontophoresis is not well known by health professionals, and it is widely used during invasive treatments, such as injection at the point of injury. This option may induce pain (the main disadvantage) or even infection. Therefore, the advantage of iontophoresis is the absence of pain during therapy application and fewer side effects, since it is a non-invasive technique. In addition, it is a safe, cheap, and effective technique for multiple pathologies with few side effects [20,21]. On the other hand, iontophoresis may have a synergistic effect in moderate or severe chronic pathologies in the short term when topical treatment is necessary, so it may be useful in combination with other conservative therapies or as a provisional treatment until the definitive biomechanical orthoses are available [22].

If conservative treatment is ineffective, extracorporeal shockwave therapy may be considered. There are different theories that have explained the mechanism of action of shockwaves, including tissue regeneration associated with neovascularization. Extracorporeal shockwave therapy, which can be divided into radial shockwave therapy (rSWT) and focused shockwave therapy (fSWT), has been widely used in clinical practice [23]. The exact effect of this therapy is not well understood. There is a non-invasive technique with a high success and efficacy rate and no unwanted effects, which is the application of acoustic impulses directed at the origin of the plantar fascia, and this is called rEWST. This therapy is on the rise in many rehabilitation and physiotherapy practices [9,10,11]. There is significant scientific evidence for the efficacy of extracorporeal shockwave therapy in the treatment of chronic PF. Extracorporeal shockwaves are a new conservative physical therapy for the treatment of musculoskeletal disorders or foot pathologies, as common as PF. There is a prevalence of 11–15% of PF with respect to other foot diseases [23], and 80% of pathologies treated with shockwaves improve considerably [24]. Since 2000, this method has been accepted in the United States as a treatment for PF. This therapy provides analgesia and stimulates the healing process by removing inflammatory debris and promoting neovascularization (the development of new blood vessels). It is recommended as a treatment for PF when other conservative treatments are not useful [25].

There are two application modalities: rESWT (radial extracorporeal shockwave therapy) and, on the other hand, focused extracorporeal shockwaves (fESWT). Focused shockwave devices electromagnetically, electropneumatically, or piezoelectrically generate energy that is transmitted to a small region of interest (focus) with the maximum energy level developing some centimeters subcutaneously. On the other hand, radial shockwave devices are pneumatically actuated, develop their maximum energy at the surface of the skin and distribute it radially in the tissue. An advantage of this method is that a larger volume of tissue can be covered. Therefore, the maximum energy is at the interface between the skin and the transducer and is reduced at the skin surface [26,27,28]. Although plantar fasciitis resolves in a short period of time, it is sometimes necessary to address personalized treatments for these patients when their evolution becomes chronic. Therefore, it is important to explore new therapies that improve the quality of life of patients with fasciitis, offering them personalized care based on scientific evidence. For this reason, it is necessary to carry out this type of research to help resolve the lack of studies on the topic, in the opinion of the authors.

Therefore, the aim of the study was to compare the effectiveness of two different conservative management approaches in the treatment of fascial inflammation-causing pain during a short period of time of 5 weeks, by comparing iontophoresis versus shockwaves, and in this way to evaluate the time of their efficiencies. This study is the first randomized controlled trial to compare the use of shockwave therapy, a widely used therapy in physiotherapy and rehabilitation clinics, with iontophoresis, a treatment for the management of plantar fasciitis. The findings will potentially provide important information and aid in clinical decision making about the performance of both devices considering the recovery time and value of both devices to improve fasciitis symptoms over a short period.

## 2. Materials and Methods

### 2.1. Study Design and Sample

This study is a randomized controlled clinical trial with a parallel group design, and it is double-blind. The groups of study were followed during 5 weeks of PF symptoms, from May 2021 to June 2022. The physician and patients were blinded to the study group assigned. For the blinding of the participants, a surgical drape was used to prevent visualization of the area to be treated. Through a sequence generated by a researcher (R.C.-M.) (http://www.random.org) (accessed on 18 March 2021), random assignment to the study groups was established. A closed envelope method was used to generate the randomization allocation sequence.

Characteristics of participants: A total sample group of 127 participants with unilateral chronic PF was recruited from the Clinical Area of the University of Seville (Spain). After inclusion in the study, the same professional with 20 years of experience conducted data collection, biomechanical foot examination, and ultrasound evaluation to check the inclusion criteria of the research to minimize the risk of measurement bias (J.A.-d.-C.).

Inclusion criteria were adult patients of both sexes with a unilateral diagnosis of PF at the inclusion moment of the study (a pain evolution of more 8 weeks and an ultrasound confirmed image of more than 3 mm thickness) confirmed by the recruiting clinician (J.A-C). The exclusion criteria were as follows: (1) previous heel surgery, fasciotomy, or heel spur surgery; (2) history of foot fracture; (3) pregnancy; (4) history of rheumatic disease; (5) neurological or vascular disease on the foot; (6) foot length discrepancy > 10 mm; (7) previous dorsal foot surgery; (8) use of foot orthoses.

All participants voluntarily agreed to participate in the investigation and, after being informed of the nature of the investigation, signed the informed consent form. The anonymity of the participants and the confidentiality of the data were guaranteed. This study protocol was approved by the Ethics Committee for Biomedical Research of the Junta de Andalucía (Ref. Approval Number: AP-2021.0118N21 on 1 December 2021) and was registered on ClinicalTrials.gov (Ref. NCT04917406). All procedures were carried out in accordance with the Declaration of Helsinki and the CONSORT statement. This study was financed by the Vice Rectorate of Research of the University of Seville (Spain). There were no conflicts of interest.

### 2.2. Intervention—Procedure

The patients were evaluated by an investigator (J.A.-d.-C.) to confirm the inclusion/exclusion criteria. After the initial assessment, patients with chronic PF were randomly assigned to the following two groups (Group A or experimental, and Group B or control). The randomization allocation of each group was carried out by a collaborating investigator (R.C.-M.) who established the sequence based on a sequence generator, in a 1:1 ratio. Group A received iontophoresis therapy, while group B received shockwave therapy. rESWT and iontophoresis therapy were applied by the same investigator (J.A.-d.-C.) [6].

The first baseline data were collected the day of the inclusion in the study; the data for age, sex, body mass index (BMI), the foot posture index (FPI), the visual analogue scale (VAS) in centimeters, and the EQ-5D or fascia thickness were measured in person at baseline [29].

The participants were included in two different groups. The iontophoresis treatment participants or group A underwent the following intervention once a week for a maximum of 5 weeks. Depending on the pain of the PF, the number of sessions was variable, although usually the maximum consisted of a total of 5 sessions. To apply this therapy, first, we degreased the skin with alcohol and waited for the area to dry completely. We assessed the absence of injury or redness at the skin level. An iontophoresis dispenser (Cardiva, Medical^®^, Santa Clara, CA, USA) using a galvanic current was used, with a two-layer capping electrode to administer the drug and a self-adhesive return capping electrode (Dupel Blue^®^, Chattanooga, Dallas, TX, USA). The medication was added to the pad and the appropriate electrode was connected, selecting the polarity. Specifically, lidocaine was added together with dexamethasone to the pad that connected to the positive electrode (anode) and the negative electrode (cathode) without medication. Lidocaine is indicated for dermal procedure analgesia, and thanks to dexamethasone iontophoresis, it benefits from the anti-inflammatory effects of dexamethasone [30]. In this sense, the risk of rupture of the plantar fascia must be taken into account as a risk factor for corticosteroid injection [31,32].

**Figure 1:**
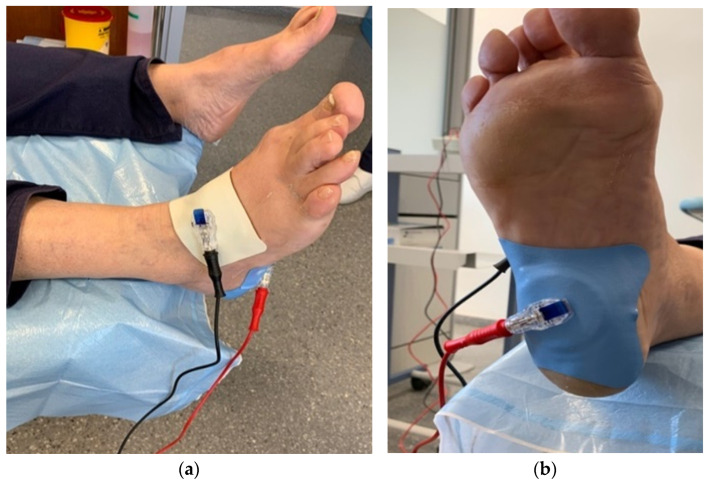
Dorsal image (**a**) and plantar image (**b**) of the application of the Cardiva^®^ iontophoresis dispenser.

Finally, the iontophoresis dosing device was programmed (Figure 1) and the dose to be applied was selected for a time of 10 min at 4 mA for each drug. This amplitude was achieved progressively until the patient felt a tingling or itching sensation. In this case, the two drugs had to be administered together on the electrode, starting with the drug with positive polarity (lidocaine 4%); after 10 min, the electrode polarity was changed (negative polarity with dexamethasone 4 mg/mL) [30,33].

**Figure 2:**
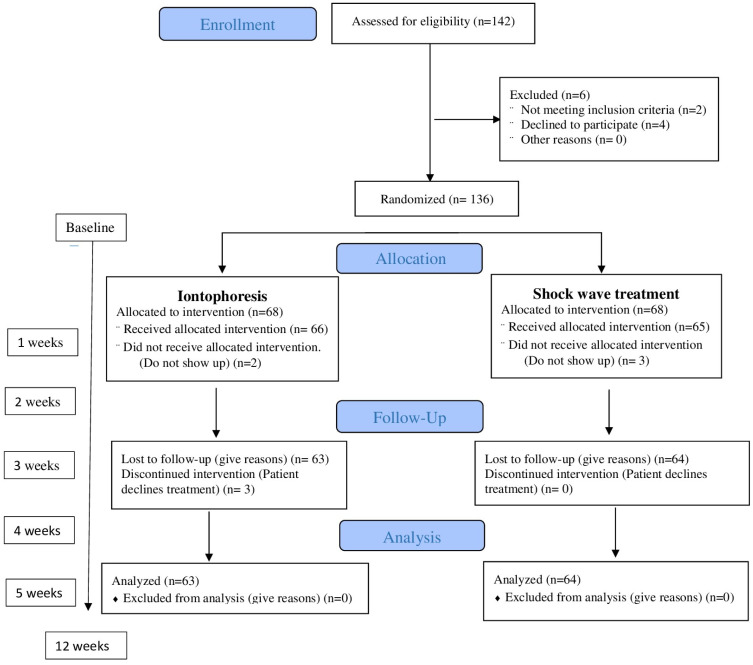
Flowchart of the progress through the phases of enrolment, intervention allocation, follow-up, and data analyses of a parallel randomized trial of two groups.

Participants assigned to group B received three sessions once a week (no more were necessary in all patients) of rESWT using a STORZ DUOLITH^®^ SD1 ultra (Storz Medical AG, Tägerwilen, Switzerland) [34,35,36]. Treatment was administered to the point of maximum sensitivity at the insertion of the medial plantar fascia in three sessions using biofeedback, with a one-week interval. A total of 2000 pulses (frequency = 5 Hz) were applied. The energy flux density was 0.20 mJ/mm^2^ [26,36]. A medium energy level (energy flux density: 0.10–0.20 mJ/mm^2^) was administered, as a recent meta-analysis recommended this as being the most efficient range for the treatment of PF [37]. The patient’s interaction with the devices in the other session was the same as in the first session. Some patients had complete pain remission in the second treatment session but were administered a total of three sessions regardless [38]. Figure 2 represents the process of enrolment, intervention allocation, follow-up, and data analyses of a parallel randomized trial of two groups. Both groups were randomly assigned (Figure 2).

### 2.3. Outcome Instruments and Variables

The primary outcome measures were as follows: the EQ-5D pain using the VAS scale and control using an imaging procedure that evaluated the final thickness of the fascia by ultrasound. The variables assessed in relation to the foot for the PF were the foot posture index (FPI) and the plantar fascia thickness.

The VAS and the fascia thickness were evaluated at each follow-up visit, with one every week until complete remission of pain; the EQ-5D was evaluated at the beginning and end of the study. The visual analogue scale (VAS) (in centimeters, cm) is a tool to evaluate numerical pain intensity from 0 (no pain) to 10 (maximum level of pain) [39], and the version of the questionnaire EQ-5D-5L is composed of five items that evaluate quality of life and the auto-self-thermometer scale. This tool evaluates five dimensions of quality of life, namely mobility, self-care, usual activities, pain/discomfort, and anxiety/depression, using a thermometer scale. It is included in a global evaluation of a person’s quality of life. The score ranges from 0 (worst health status) to 100 (best health status), and it is computed by measuring the distance (in mm) between the end of the line marked with “the worst health you can imagine” and the mark on the line indicated by the patient.

The foot posture index (FPI) quantifies [40], meanwhile, six items, namely the morphology and position (supinated, pronated, or neutral) of the foot. The summative evaluation of six criteria defines said position. The values can be as follows: pronated foot position (+6 to +12), neutral position (0 to +5), and supinated position (12 to 1), taking into account that each criterion evaluates from 2 to +2 (range 12 to +12). The six criteria are as follows: talar head palpation, supramalleolar and inframalleolar curvature, frontal plane position of the calcaneus, talonavicular prominence, medial arch congruence, and forefoot abduction/abduction [40].

Another questionnaire carried out at the end of the process was the PGI-I (Patient Global Impression of Improvement) questionnaire, which evaluates the improvement reported by the patient after the application of the two therapies. This evaluation was performed after 12 weeks of treatment [41]. The patient’s perception of treatment efficacy was measured using the Patients’ Global Impression of Change (PGIC), consisting of a 7-point scale (very much improved, much improved, minimally improved, no change, minimally worse, much worse, and very much worse) [42].

The Alpinion^®^ E-CUBE-7 ultrasound machine (Medical Scan, Seoul, Republic of Korea) with a high-frequency linear translator (L3-12 MHz) was used for fascial ultrasound examination. Following previous studies, the investigators who performed the measurements were trained in plantar fascia ultrasound and were unaware of the modality of treatment and the assignment of the group. Each measurement was taken by the same physician (E.B.-B) and was taken as the mean value of both measurements. Following the established protocol, the patients were examined on a stretcher in a prone position with the foot hanging, the knee extended, and the ankle in a 90° dorsiflexed position. The probe was placed on the plantar aspect of the hindfoot, and measurements were taken from the same anatomical landmarks [43,44]. The thickness of the plantar fascia was measured at the 5 mm insertion of the plantar fascia from the distal tuberosity of the medial calcaneus [45], and each patient was measured six times. PF was diagnosed when the patient presented with a fascia thickness greater than 3 mm and clinical pain throughout the course of the fascia.

### 2.4. Sample Size and Statistical Analyses

The sample size was determined with the G-Power 3.1.0 software, which was used to calculate the study sample size^®^ (Franz Faul, Universität Kiel, Germany). Changes in effect size greater than 0.5 and measurements between independent samples were compared; those having possible type I and II errors of 0.05 and 0.2, respectively, were taken into account in this study. The minimum sample size needed was 102 participants, but that number was increased by 35% to compensate for possible losses of participants.

The IBM SPSS^®^ 23.0 Version software package for Windows (IBM, Armonk, NY, USA) and Excel were used for statistical analysis. Numerical (quantitative) variables are summarized with means and standard deviations (mean ± SD) or, if highly skewed, medians and percentiles (P25 and P75), while nonnumerical (qualitative) variables use frequencies and percentages. The Kolmogorov–Smirnov test was used to determine the normal distribution of the variables.

Descriptive statistics were performed for the variables age, sex, BMI, VAS, and FPI baseline. The Student’s *t*-test was used for comparison between group means in both VAS and fascia thickness. When a normal distribution pattern was rejected, the nonparametric Mann–Whitney U test was applied. Furthermore, a paired *t*-test analysis was performed to assess differences between groups. The secondary results were assessed for differences using Chi-square tests for qualitative variables.

A repeated measures ANOVA test was carried out to evaluate the evolution over time of the treatment with respect to pain analysis (VAS). When a significant effect over time was detected, Bonferroni post hoc analysis with multiple comparison correction was used for comparisons in pairs among the different assessment times. The MCID has been calculated by the anchor-based method, taking into account the patient’s perception of clinical improvement and the clinical data of fascia (less than 3 mm indicates non-inflammatory process) and pain (reference value 4 according to VAS scale).

The analysis was performed with the intention of treating, and a value of *p* < 0.05 was established as being statistically significant.

## 3. Results

### Sociodemographic and Clinical Characteristics of the Sample

A total sample group of 142 patients was initially recruited, but only 127 participants finished the study (15 dropped out of the research because they did not attend the appointment or declined follow-up treatment, preferring manual therapy). The baseline characteristics at the beginning of the study are shown in Table 1. The baseline conditions of the two groups and the whole sample were analyzed. There was parity in sex (44.1% women and 55.9% male), the mean age was 50.1 ± 10.3 years, and the BMI values were situated at 27.1 ± 2.4.

Homogeneity was observed at a baseline between the baseline of the iontophoresis group with respect to the shockwave group for all study variables (*p* > 0.05).

An analysis of the PF pain (VAS) and the final thickness of the fascia after complete remission of symptoms was carried out. The results are shown in Table 2. A significant difference in pain was observed in all measurements. If we perform the individual evaluation by treatment, significant changes in iontophoresis were observed during all measurements. In the case of shockwaves, there were no significant changes in the last session, since the pain had completely subsided.

Regarding iontophoresis, data analysis revealed that the cut-off point of 5.0 on the VAS scale and 2.05 millimeters on the fascia corresponded to the MDMI in our sample. This cut-off point demonstrated a sensitivity of 22% and a specificity of 39%, indicating that it may not be optimal for accurately identifying cases with a value of 5 or more on the VAS. However, the sensitivity and specificity was 100% for fascia (2.05 on average) and this was considered clinically significant for our patients.

On the other hand, regarding the analysis of the ESWT group, the data revealed that the cut-off point of 6.0 on the VAS scale and 3.10 millimeters on the fascia corresponded to the MDMI in our sample. This cut-off point demonstrated a sensitivity of 100% and a specificity of 100%, both at the fascia level and in the pain analysis. These were the minimum clinically relevant differences for the patients.

After performing the post hoc test to assess intra-group comparisons across sessions, it was observed that iontophoresis showed significant differences in all measurements (≤0.0001), with no such difference found in the shockwave treatment between sessions 4 and 5 (*p* = 0.999).

Regarding the overall impression of the patient, this is better in the shockwave group (83.1% vs. 37.1%), although in both groups it is quite good (Table 3).

In Table 4, an analysis is made of the quality of life of the subjects before and after the treatments. Improvements are observed in the iontophoresis and shockwave groups in all areas (Table 4).

**Figure 3:**
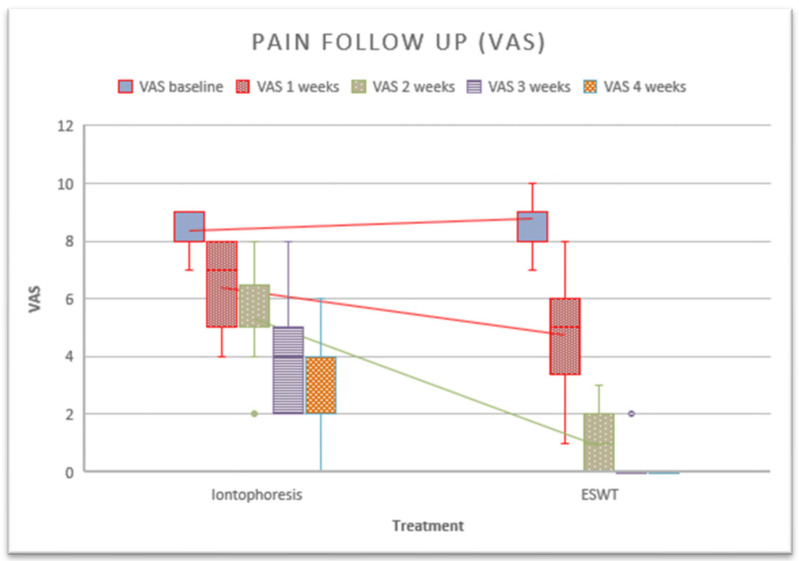
Follow-up of pain after the application of treatments.

The main results of this study showed significant differences between the groups with respect to pain after the third treatment session in favor of the rESWT group (Figure 3). Using within-group analysis, there were statistically significant improvements in the measures of self-reported pain when comparing data at baseline to those at follow-up for members of the intervention and control groups after 5 weeks. On the other hand, a paired sample analysis was performed to assess whether there were differences between measurements. Differences were found in the iontophoresis group between the baseline session and the second session (*p* = 0.001), between the second session and the third session (*p* = 0.001), between the third and fourth sessions (*p* ≤ 0.001), between the fourth and fifth sessions (*p* = 0.001). In the rESWT group, a difference was observed between the first and second sessions (*p* ≤ 0.001), as well as between the second and third sessions (*p* ≤ 0.001).

## 4. Discussion

The aim of this double-blind clinical trial was to compare the effectiveness of conservative treatment of PF with a dual approach, namely iontophoresis and shockwaves as a physical treatment for PF in a short time period of 5 weeks. The results of this study may indicate that shockwave therapy is more effective than iontophoresis in the treatment of PF, as the scientific evidence showed. Despite this fact, we consider new treatment approaches to be useful when the available arsenal is not effective, or if we need a quick improvement in symptoms due to the person’s disability with PF. Our findings revealed some indications of the usefulness of shockwave therapy in relation to pain when compared to iontophoresis. The rESWT effect improved the symptoms from the third session onwards and offered significant differences. Some of the most widely described tools in the literature [46], such as the VAS, have been used to assess shockwave follow-up.

Regarding the mechanism of action of shockwaves, there is no clear consensus on the mechanism of action, although its benefits, such as vascularization, protein biosynthesis, and pain reduction, among others, stand out. Shockwaves transport energy to painful points and allow tissue regeneration. Furthermore, thanks to the overstimulation of that pain area and after different processes, such as the alteration of the permeability of the cell membrane and its cellular activity, a reduction in pain occurs [47,48]. This energy spreads through the tissues and completes the improvement in the functionality of said tissues and in this case the plantar fascia [48,49]. These approaches reinforce the existing discrepancies in this treatment modality and were the subject of this study. Grecco et al. 2013 and Razzano et al., 2017 recommend shockwave treatment for chronic PF after a refractory response to conservative care and just before surgery [50,51].

Traditionally, patient pain has been subjectively assessed, as demonstrated by most of the studies consulted [52]. PF is a very prevalent and disabling musculoskeletal condition that requires effective treatments to reduce the pain and limitation of daily activities in patients, so that healing can take place as quickly as possible. Regarding shockwave therapy, in 2018, the study by Vaamonde-Lorenzo et al. [53] on the treatment of PF with piezoelectric focal shockwaves showed that patients showed an improvement in pain in the medium term (3 months), which is an effective and satisfactory treatment for patients. Systematic reviews have shown satisfactory short-term pain relief and functional outcomes, although long-term efficacy remains unknown because of a lack of long-term data [10,54].

In 2020, Elía-Martínez et al. [52] compared the effectiveness of fESWT and rESWT; they found no differences in a non-randomized sample of 69 patients in their measurements (VAS, plantar fascia thickness measured by ultrasound, Roles and Maudsley scale) at 3 months. Based on evidence from some systematic reviews in physical therapy, there is no consensus on the difference between low-energy and high-energy shockwave therapy devices [53]. In 2021, a systematic review of clinical trials on the efficacy of shockwave therapy in PF included 11 RCTs with 658 patients and concluded that there were beneficial results in pain and function in these patients [53]. Other studies indicate that shockwaves produce a vibration that affects ankle application and could improve the functionality produced by PF, secondary to an increase in proprioception [55]. According to the literature, shockwaves were found to be an effective treatment in PF, if combined with other treatment modalities [56].

Coheña-Jiménez et al. [57] conducted an RCT with 83 patients comparing shockwave therapy together with custom-made insoles versus placebo insoles. Their results showed reductions in pain and improvements in function in combined therapy with custom plantar supports, with patient perceptions being good (85%) and excellent (97.5%) in the medium term. Other hypotheses suggest the need for global postural rebalancing [58]. Recently, in 2022, a study evaluated the efficacy of shockwave therapy on myofascial points, combining concepts of myofascial manipulation and shockwave therapy. This study is in line with myofascial impairment in the painful heel area secondary to biomechanical imbalance of the foot and lower extremity. Patients showed improved FFI scores after treatment, and at 1 month and 4 months of follow-up. According to these authors, the efficacy of the treatment would be in the global biomechanical rebalancing of the body, obtaining better results in improvements in the pain, disability, and recovery time of the myofascial group, but the limitations of the study were the low number of participants and the short follow-up period [58].

In contrast, some studies report that shockwave therapy was effective when other therapies failed [2,26], with only minor results [59], but improvements in function were found in most cases. Numerous treatments for PF have been described in the literature, both in isolation and in combination, but not all are effective. To our knowledge, this is the first study to compare these different treatments for plantar pain, specifically shockwave therapy versus iontophoresis. In addition, to monitor the effectiveness of the shockwaves, measurements of the thickness of the plantar fascia were taken before the first session was applied.

Previous studies have measured plantar fascia thicknesses, measured with ultrasound, after shockwave treatment, and these concluded that the clinical differences were insignificant. However, these authors reported that the thinner plantar fascia was less painful and observed minimal reductions in plantar fascia (0.4 mm; *p* < 0.05) in patients receiving low-intensity shockwave treatment [60]. This is consistent with most authors noting significant decreases in plantar fascia thickness, but contrary to that reported by Lai et al. [61] 2018 who observed increases in plantar fascia thickness 4 weeks after shockwave treatment (*p* = 0.048). Lai et al. [61] observed an increase in plantar fascia thickness in the first month and a significant decrease in the third month after shockwave treatment from 0.37 ± 0.07 to 0.46 ± 0.08 cm.

Previous studies have investigated iontophoresis with dexametaxone as a treatment for musculoskeletal pathologies [62]. The study by Cleland et al. [46] observed clinical improvement with reduced pain and increased function in a group of 30 patients with PF who were treated with lateral iontophoresis with dexamethasone, stretching of the gastrocnemius muscle, and the application of ice at the end of the session. In addition, these patients received ultrasound therapy (3 MHz, 1.5 W/cm^2^, and 100 Hz) to improve skin permeability and better introduce dexamethasone (40 mA-min). Other studies have compared the effectiveness of iontophoresis combined with low-dye bandaging [63]. In one study, RCT 31 patients (42 feet) with PF were treated for 2 weeks with six sessions and continuous taping, and received dexamethasone (0.4%), placebo (NaCl 0.9%), and acetic acid (5%). These authors reported better results with a greater reduction in pain and stiffness in the morning in the group that received acetic acid sessions. In contrast, our findings in the treatment of PF showed progressive differences in pain between treatment sessions.

Finally, it was observed that mitigating pain substantially improves the quality of life for patients (mobility dimensions, activities of daily living, and self-care, among others) [63]. This is in line with the results recently contributed by Cotchett in 2022 [55], where they evaluated the impact of foot pain on the mental health of individuals. After performing the systematic review, they found a significant association between emotional distress with foot pain and foot function in some people with plantar heel pain. In addition, kinesiophobia and pain catastrophizing were significantly associated with impaired foot function. The authors conclude that negative psychological constructs are higher in participants with foot pain [64].

We believe that the findings of this study may help to improve the treatment of this condition and that iontophoresis may be included as another conservative treatment for FP. The chronicity of the symptoms of plantar fasciitis and the patient’s expectations can generate a positive response trend in reducing pain. A bias may arise secondary to the treatment modality, given that many patients prefer to undergo non-invasive treatment rather than plantar fasciitis surgery. The benefits found in this study are consistent with other research suggesting that although improvement in pain and stiffness is the most valued benefit in studies, it is important to keep in mind that there are other personal characteristics that can affect healing. This study adds to the body of knowledge on iontophoresis therapy. It is an inexpensive therapy that would allow clinicians to increase their therapeutic arsenal with a lower initial investment power compared to shockwave devices. Our knowledge is based on a short-term assessment, but an important aim of this study was to evaluate if PF could improve quality of life in a short period, even provisionally while the subject awaits other more definitive treatment, such as rehabilitation with stretching or customized foot orthoses.

The main limitation of this work has been the short-term evaluation; this issue has been justified because both therapies shown do not modify the function of the foot, indicating that they are initial treatments, rather than being definitive. Although it is true that the follow-up of the treatment took place at 3 months, this was only assessed by means of the patient’s impression scale and their improvements on the EQ 5D thermometer.

It would also have been interesting to find out more about the lifestyle patterns of the patients or whether they had previous experience with plantar supports or physical therapy. On the other hand, studies showing longer-term outcomes and analyzing possible recurrence rates are recommended. Furthermore, the cost of both therapies should be taken into account, both in terms of the consumables required and the cost of both devices.

## 5. Conclusions

Shockwave therapy offers better effectiveness than iontophoresis therapy for the treatment of PF in a short-term period of 5 weeks, offering improvements in pain reduction from the first session, and improving and maintaining that improvement, which is statistically significant. The decrease in plantar fascia thickness at the end of treatment was correlated with a decrease in pain scores. Both iontophoresis (lidocaine together with dexamethasone) and shockwave therapy offer benefits for the treatment of PF, but studies assessing the cost–benefit are needed. It should be noted that iontophoresis devices are less expensive than shockwave equipment, hence the need to include the cost variable as well as recovery times.

## Figures and Tables

**Table 1:** Sociodemographic and clinical characteristics of the treatment groups.

Characteristic		Total N (%) (N = 127)	Experimental Group A N (%) (N = 63) (Iontophoresis)	Control Group B N (%) (N = 64) (rESWT)	*p*-Value
Sex ^b^	Female	56 (44.1)	26 (41.9)	30 (46.2)	0.632
	Male	71 (55.9)	36 (58.1)	35 (53.8)	
FPI ^b^	Pronation	95 (74.8)	49 (78.0)	46 (70.8)	0.139
	Highly pronated	17 (13.4)	9 (14.6)	8 (12.3)	
	Neutro	5 (3.9)	0 (0.0)	5 (7.7)	
	Supinated	10 (7.9)	4 (7.3)	6 (9.2)	
BMI (Kg/m^2^) ^c^ Mean ± SD		27.1 ± 2.4	27.0 ± 2.4	27.1 ± 2.3	0.896
Age (years) ^a^ Mean ± SD		50.1 ± 10.3	51.5 ± 11.6	48.8 ± 8.7	0.141
Initial thickness fascia (mm) ^c^ Mean ± SD		4.2 ± 0.8	4.2 ± 0.7	4.3 ± 0.4	0.521
VAS baseline ^c^ Mean ± SD		8.6 ± 0.8	8.5 ± 0.7	8.7 ± 0.8	0.174
EQ-5D (thermometer scale) ^c^ Mean ± SD		30.5 ± 8.5	29.4 ± 7.7	31.61 ± 9.0	0.133

BMI: body mass index; rESWT: radial extracorporeal shockwave therapy; SD: standard deviation. ^a^ Mann–Whitney U test. ^b^ Chi-square test. ^c^ Student’s *t*-test.

**Table 2:** Evaluation of the variable VAS between Group A (the experimental group) and Group B (the control group) with respect to the follow-up period of 5 weeks and results relative to the effects over time with the post hoc analyses outputs, as well as changes in fascia thickness and the EQ-5D thermometer scale.

VAS (N = 127)		T_0_	T_1_	T_2_	T_3_	T_4_	T_5_	Fascia Thickness T_4_	EQ-5D (Thermometer Scale) T_5_
Experimental Group A (N = 63)	Mean ± SD	8.5 ± 0.7	6.5 ± 1.6	5.1 ± 1.7	3.8 ± 1.5	2.5 ± 1.4	2.4 ± 1.2	3.4 ± 0.6	56.2 ± 10.3
	95%CI	8.3–8.7	6.1–6.9	4.7–5.5	3.4–4.2	2.2–2.9	2.0–2.6	3.2–3.6	53.6–58.9
MCDI		-	-	5.50	-	-	-	2.05	-
Control Group B (N = 64)	Mean ± SD	8.7 ± 0.8	4.8 ± 1.6	1.0 ± 0.9	0.1 ± 0.3	0.0 ± 0.0	0.0 ± 0.0	3.0 ± 0.2	67.0 ± 9.4
	95%CI	8.5–8.9	4.4–5.2	0.8–1.2	0.0–0.1	0.0–0.0	0.0–0.0	3.0–3.1	64.7–69.3
MCDI		-	-	6.0	-	-	-	3.10	-
Overall significance in time	*p*-value	0.174	≤0.0001 ***	≤0.0001 ***	≤0.0001 ***	≤0.0001 ***	≤0.0001 ***	≤0.0001 ***	≤0.0001 ***
Experimental Group A (N = 63) comparison in pair-significance		T_0_ vs. T_1_, T_2_, T_3_, T_4_, T_5_	T_1_ vs. T_0_, T_2_, T_3_, T_4_, T_5_	T_2_ vs. T_0_, T_1_, T_3_, T_4_, T_5_	T_3_ vs. T_0_, T_1_, T_2_, T_4_, T_5_	T_4_ vs. T_0_, T_1_, T_2_, T_5_	T_5_ vs. T_0_, T_1_, T_2_, T_4_	-	-
Group B (N = 64) (ESWT) comparison in pair-significance		T_0_ vs. T_1_, T_2_, T_3_, T_4_, T_5_	T_1_ vs. T_0,_ T_2_, T_3_, T_4_, T_5_	T_2_ vs. T_0_, T_1_, T_3_, T_4_, T_5_	T_3_ vs. T_0_, T_1_, T_2_, T_5_	T_4_ vs. T_0_, T_1_, T_2_, T_3_	T_5_ vs. T_0_, T_1_, T_2_, T_3_	-	-

rESWT: Radial extracorporeal shockwave therapy; SD: standard deviation; CI: confidence interval. MCID: minimum clinically important difference. Significance set at *p* < 0.05. *** *p* < 0.001. T_0_: Baseline, T_1_: second session, T_2_: third session, T_3_: fourth session, T_4_: fifth session, and T_5_: assessment at 12 weeks.

**Table 3:** Scores on the Patients’ Global Impression of Change scale in Group A and Group B.

	Very Much Improved	Much Improved	Minimally Improved	No Change	Minimally Worse	Much Worse	Very Much Worse	*p*-Value
PGIC-I ^T4^ (Iontophoresis)	23 (37.1)	24 (38.7)	15(24.2)	0 (0.0)	0 (0.0)	0 (0.0)	0 (0.0)	≤0.0001 ***
PGIC-I ^T4^ (ESWT)	54 (83.1)	11 (16.9)	0 (0.0)	0 (0.0)	0 (0.0)	0 (0.0)	0 (0.0)	

PGIC-I: Patients’ Global Impression of Change. Significance set at *p* < 0.05. *** *p* < 0.001. ^T4^: Fifth session.

**Table 4:** Evaluation of the questionnaire EQ-5D, for the total sample, and a comparison between Group A (experimental group) and Group B (control group) for the follow-up period.

EuroQol-5D Items	Experimental Group A (IONTOFORESIS)			Control Group B (rESWT)		
	Baseline N (%)	Endpoint N (%)	*p*-Value	Baseline N (%)	Endpoint N (%)	*p*-Value
EuroQol-5D/Mobility						
1. I have no trouble walking.	10 (15.9)	58 (92.1)	*p* < 0.001 ^c^	10 (15.6)	54(84.4)	*p* < 0.001 ^b^
2. Some difficulty walking.	42 (66.7)	5 (7.9)		45 (70.3)	10 (15.6)	
3. I have to stay in bed.	11 (17.5)	0 (0)		9 (14.1)	0 (0)	
EuroQol-5D/Self-care						
1. I have no issues with self-care.	15 (23.8)	53 (84.1)	*p* < 0.004 ^b^	17 (26.5)	60 (93.8)	*p* < 0.001 ^c^
2. Some difficulty with self-care.	44 (69.8)	9 (14.3)		43 (67.2)	4 (6.2)	
3. I am unable to perform self-care.	4 (6.3)	1 (1.6)		4 (6.3)	0 (0)	
EuroQol-5D/Daily activity						
1. I have no issues with daily activities.	5(9.4)	47 (74.6)	*p* < 0.023 ^b^	12 (18.8)	51 (79.7)	*p* < 0.001 ^b^
2. Some difficulty with daily activities.	56 (87.5)	16 (25.4)		49 (81.2)	13 (20.3)	
3. I am unable to perform daily activities.	2 (3.1)	0 (0)		3 (0)	0 (0)	
EuroQol-5D/Pain						
1. No pain or discomfort.	0 (0)	41 (65.1)	*p* < 0.004 ^c^	0 (0)	56 (87.45)	*p* < 0.001 ^c^
2. Moderate pain or discomfort.	33 (52.4)	19 (30.2)		30 (46.9)	7 (10.9)	
3. Severe pain or discomfort.	30 (47.6)	3 (4.8)		34 (53.1)	1 (1.6)	
EuroQol-5D/Anxiety						
1. Not anxious or depressed.	33 (52.4)	47 (74.6)	*p* < 0.001 ^c^	15 (23.4)	35 (54.7)	*p* < 0.006 ^b^
2. Moderately anxious or depressed.	21 (33.3)	9 (14.3)		33 (51.6)	19 (29.7)	
3. Very anxious or depressed.	9 (14.3)	7 (11.1)		16 (25.0)	10 (15.6)	

χ^2^ = Chi-square test; ^b^ medium effect size (30 < ᶲ ≤ 50); ^c^ large effect size (ᶲ ≥ 50).

## Data Availability

Data are available by contact with the correspondence authors.
